# Mal-Lys: prediction of lysine malonylation sites in proteins integrated sequence-based features with mRMR feature selection

**DOI:** 10.1038/srep38318

**Published:** 2016-12-02

**Authors:** Yan Xu, Ya-Xin Ding, Jun Ding, Ling-Yun Wu, Yu Xue

**Affiliations:** 1 Department of Information and Computer Science, University of Science and Technology Beijing, Beijing 100083, China; 2 Institute of Applied Mathematics, Academy of Mathematics and Systems Science, Chinese Academy of Sciences, Beijing 100190, China; 3 Department of Biomedical Engineering, College of Life Science and Technology, Huazhong University of Science and Technology, Wuhan, Hubei 430074, China

## Abstract

Lysine malonylation is an important post-translational modification (PTM) in proteins, and has been characterized to be associated with diseases. However, identifying malonyllysine sites still remains to be a great challenge due to the labor-intensive and time-consuming experiments. In view of this situation, the establishment of a useful computational method and the development of an efficient predictor are highly desired. In this study, a predictor Mal-Lys which incorporated residue sequence order information, position-specific amino acid propensity and physicochemical properties was proposed. A feature selection method of minimum Redundancy Maximum Relevance (mRMR) was used to select optimal ones from the whole features. With the leave-one-out validation, the value of the area under the curve (AUC) was calculated as 0.8143, whereas 6-, 8- and 10-fold cross-validations had similar AUC values which showed the robustness of the predictor Mal-Lys. The predictor also showed satisfying performance in the experimental data from the UniProt database. Meanwhile, a user-friendly web-server for Mal-Lys is accessible at http://app.aporc.org/Mal-Lys/.

Post-translational modifications (PTMs) play crucial roles in various cell functions and biological processes, as well as in regulating cellular plasticity and dynamics. Among the 20 types of natural amino acids occurred in proteins, lysine is one of the most heavily modified residues^1^,^2^. Recent discoveries of multiple types of new protein lysine acylations, such as malonylation, succinylation, and glutarylation, have greatly expanded our understanding of the types of protein PTMs^1^,^3^,^4^,^5^,^6^,^7^,^8^,^9^. Because malonyl, succinyl and glutaryl groups contain a negatively charged carboxyl group, the three types of acidic lysine modifications are structurally similar and have the potential to regulate different proteins in different pathways^5^. It is also confirmed that malonylation, succinylation, and glutarylation of lysine residues are evolutionarily conserved and dynamic under diverse biological and cellular conditions, such as stress response, metabolisms, and genetic mutations^10^,^11^.

In 2011, lysine malonylated substrates were firstly identified though a high-throughput proteomic analysis, while the results demonstrated that malonyllysine in proteins is present and conserved in both eukaryotic and prokaryotic cells^8^. However, its potential functions and roles associated with human diseases remain largely unknown. A recent study characterized that lysine malonylation regulates the glycolytic flux by modifying mouse glyceraldehyde 3-phosphate dehydrogenase (GAPDH) at K184 to inhibit its enzymatic activity^3^. Also, using the liver tissues of *db*/*db* and *ob*/*ob* mice, it was observed that malonylation plays a potential role in type 2 diabetes, whereas further bioinformatic analysis of the proteomic results revealed the enrichment of malonylated proteins in metabolic pathways, especially the pathways of glucose and fatty acid metabolisms^4^.

In view of the potential importance of malonylation, identifying the malonylated sites in proteins is extremely urgent and may provide useful information for biomedical research. However, the identification and investigation of the malonylated sites are desirable and mainly depend on mass spectrometry which is expensive and laborious. As a complement for experiments, a computational method for timely and effectively identifying the malonyllysine sites is necessary when facing multitudinous protein sequences generated in the post-genomic age.

In this article, a new computational method of Mal-Lys which predicts malonyllysine sites from protein primary sequences is proposed. Amino acid position information has been succeeded in PTM prediction and achieved satisfying results^12^,^13^. Sequence order information (*k*-grams^14^), position-specific amino acid propensity and physicochemical properties (AAIndex^15^) were utilized to construct features. The algorithm of support vector machines (SVMs) was used for training the computational model, whereas the leave-one-out validation and 6-, 8- and 10-fold cross-validations were adopted to evaluate the prediction accuracy and robustness of Mal-Lys. The satisfying performance suggested that Mal-Lys can be a useful tool to identify potential lysine malonylation sites in proteins for further experimental consideration.

## Results and Discussion

### The construction of feature vectors

Totally, 494 non-redundant malonyllysine sites were collected from a previously reported large-scale study^4^. The detailed processing of the dataset was shown in Methods. Then the position specific amino acid propensity and sequence order information were utilized to convert peptide fragments into mathematical expressions for the feature construction. A peptide was denoted as


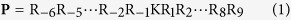


where R_*i*_ can be any of the 20 native amino acids or the dummy code X.

The *k*-grams^14^ feature construction was utilized to generate features. A *k*-gram is simply a pattern of *k* consecutive letters which could be amino acid symbols or nucleic acid symbols. We used the basic and the position-specific *k*-grams (*k* is the length of an amino acid sequence to be generated). Since there are 21 possible letters (20 native and 1 dummy amino acid) for each position, there are 21^*k*^ possible basic *k*-grams for each value of *k*. We used the basic *k*-grams feature generation with *k* = 1, 2 and got 21^1^ + 21^2^ = 462 dimensions.

Another position-specific *k*-grams simply records which *k*-gram appears in a particular position in the sequence segment. We consider only 1-gram, that is, *k*-grams for *k* = 1. Since each segment has 6 up-stream and 9 down-stream amino acids flanking each side of the target lysine (K), there are 15 position-specific 1-grams. Here, let us use the numerical codes 1, 2, 3, …, 20 to represent the 20 native amino acids according to the alphabetic order of their single letter codes, and use 21 to represent the dummy amino acid *X*.

Each amino acid has its own specific physicochemical and biologic properties which have direct or indirect effects on protein properties. AAIndex^15^ is a database which contains various physicochemical and biologic properties of amino acids. In this work, 14 properties were selected from AAIndex database, including hydrophobicity, polarity, polarizability, solvent, accessibility, net charge index of side chains, molecular weight, PK-N, PK-C, melting point, optical rotation, entropy of formation, heat capacity and absolute entropy which have shown an excellent predicted performance in the prediction of protein pupylation sites^16^. For the pseudo amino acid *X*, it was defined 0 as its physicochemical property value. Therefore, each amino acid was constructed into 14 features through AAIndex database. For a peptide fragment, a 210-D (15*14 = 210) feature vector was obtained through AAIndex encoding scheme.

Combining the three features each sequence segment is encoded into a 21^1^ + 21^2^ + 15 + 210 = 687 dimensional vector. The 35 features which are the same values in malonylated and non-malonylated peptides were been deleted. The peptide was encoded into a 652 (687–35) dimensional vector.

The post probability SVMs algorithm was implemented in LIBSVM^17^, a public and widely used SVM library. The kernel function was RBF (Radial Basis Function) kernel with the parameter g = 0.0125. For a query peptide **P** as formulated by feature construction, suppose Pr(y = 1|**P**) is its probability to the malonylated peptides. Thus, the prediction rule for the query peptide **P** can be formulated as.


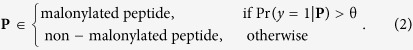


The cutoff value θ is 0.5 for balancing the true positive and negative rate. The predictor established via the above procedures is called Mal-Lys, where “Mal” for “malonylation”, and “Lys” for lysine residue.

### The evaluation of the prediction performance and accuracy

In statistical prediction, the following three cross-validation methods are often used to evaluate a predictor for its effectiveness in practical application: independent test, subsampling or *k*-fold (such as 6-, 8-, or 10-fold) cross-validations and the LOO validation. The LOO validation has been widely used in the performance evaluation of PTM site prediction^18^,^19^ for its unique result. In this work, we used the LOO validation and 6-, 8- and 10-fold cross-validations to evaluate the accuracy and robustness of the proposed predictor Mal-Lys.

**Figure 1:**
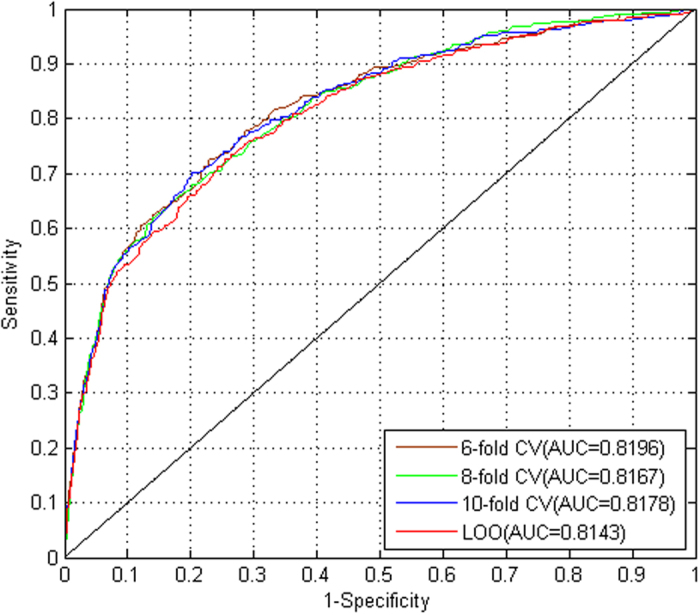
The ROC curves and their AUC values for the LOO validation and 6-, 8- and 10-fold cross-validations on the training dataset.

There were 652 features in the encoding schemes and some of them were redundancy. In this study, the mutual method of minimum Redundancy Maximum Relevance (mRMR) was applied to select features (http://penglab.janelia.org/proj/mRMR/)^20^,^21^,^22^,^23^. We selected 50 features which had the maximum relevance to the classifier and minimum redundancy to the former features. The 6-, 8- and 10-fold cross-validations had been done for 30 times and the average values were calculated. The receiver operating characteristic (ROC) curves were drawn and the area under the curve (AUC) values were also calculated (Fig. 1). In the 6-, 8- and 10-fold cross-validations, the AUC values were 0.8196, 0.8167 and 0.8178, respectively. They are similar to the LOO AUC value 0.8143 which illustrated the performance and robustness of the predictor Mal-Lys.

**Figure 2:**
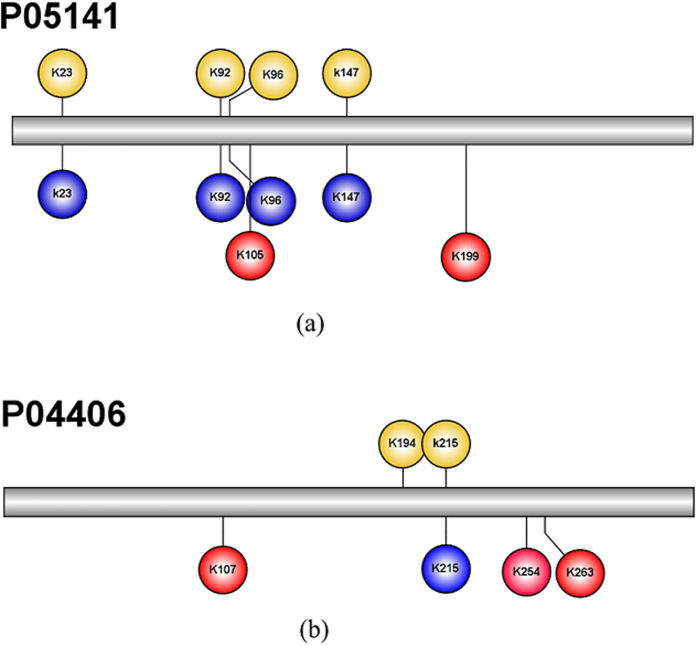
The prediction results of two human malonylated proteins, including (**a**) SLC25A5 (P05141) and (**b**) GAPDH (P04406). The experimentally identified malonyllysine sites were shown in yellow, whereas predicted results consistent with known sites were shown in blue. Newly predicted sites with high potentials to be real malonylation sites were marked in red.

To illustrate the performance of Mal-Lys, we collected experimental lysine malonylation sites including 33 malonyllysine sites (25 *Human*, 5 *Bovine*, 2 *E.coli*, 1 *A. thaliana*) from UniProt database (http://www.uniprot.org/). These 25 human lysine malonylation sites were used as the independent test. The AUC was 0.7935 which also showed the performance of the predictor Mal-Lys. The predicted results of human solute carrier family 25 member 5 (SLC25A5, UniProt accession: P05141) and GAPDH (P04406) have been plotted with IBS software^24^ (Fig. 2). Previously, SLC25A5 was experimentally identified to be malonylated at K23, K92, K96 and K147^8^ (Fig. 2a). Mal-Lys not only correctly predicts all four sites as positive hits, but also predicts two additional sites of K105 and K199 to be potential malonylation sites with high confidence (Fig. 2a). For human GAPDH, two lysine residues K194 and K215 was experimentally identified as real malonylation sites, whereas Mal-Lys can predict K215 as a positive hits. Newly predicted sites of K107, K254 and K263 can be useful candidates for further experiments.

### Feature analysis

**Figure 3:**
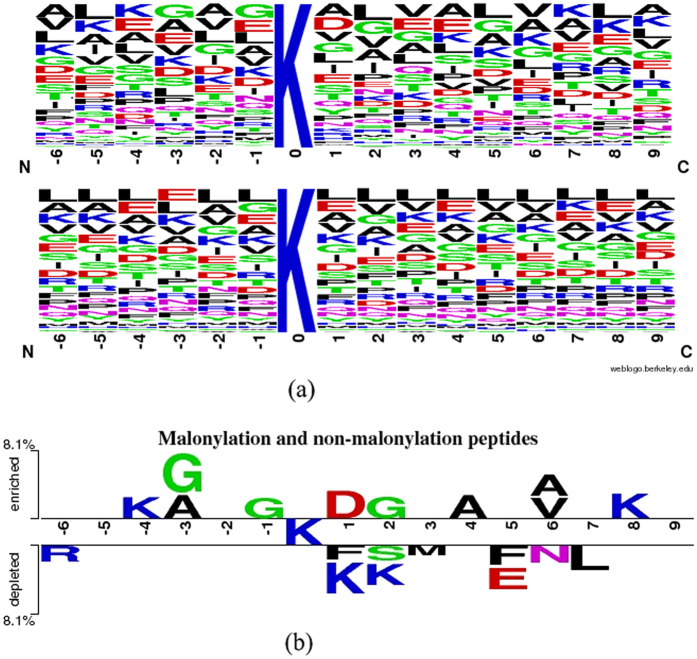
The sequence preferences of malonylation and non-malonylation peptides. (**a**) The amino acid residue frequency of positive and negative peptides on the experimentally data which contained 458 malonyllysine and 3,974 non-malonyllysine peptides. (**b**) The results of Two Sample Logo for malonylation and non-malonylation peptides with t-test (*p*-value < 0.05).

Sequence occurrence frequency on every position was utilized in the feature construction. From the sequence Logo of the experimental 458 positive malonylysine peptides and 3,974 negative non-malonylysine peptides^25^ (Fig. 3a), we found that there were not significantly statistical difference between malonylation and non-malonylation peptides. Using another web-based tool of Two Sample Logo with the t-test (*p*-value < 0.05)^26^, we observed that malonylation and non-malonylysine peptides have considerably difference sequence preferences (Fig. 3b). The polar amino acid glycine (G) was enriched at position −3, −1 and +2 in malonylation peptides, while basic lysine (K) was enriched at position +1, +2 and +8 in non-malonylation peptides. These differences in malonylation and non-malonylation peptides may improve the performance of the classifier.

### The online web-service of Mal-Lys

**Figure 4:**
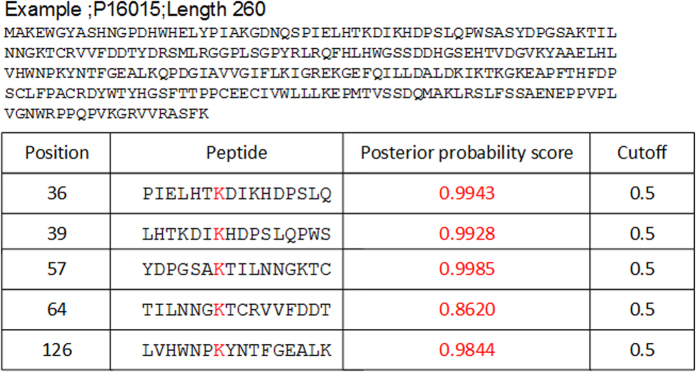
The output of the online predictor Mal-Lys. A mouse malonylated substrate, Carbonic anhydrase 3/Ca3 (P16015), was chosen as an example.

For the convenience of the vast majority of experimental scientists, a user-friendly and publicly accessible web-server is one of the keys in developing a practically useful prediction method. In view of this, we have developed a web-server for the Mal-Lys predictor in JAVA. The web-server for Mal-Lys can be freely accessible at http://app.aporc.org/Mal-Lys/. One or multiple protein sequences should be input in the FASTA format, and the output results will be shown in a tabular format (Fig. 4).

## Conclusion

As a newly discovered PTM, lysine malonylation has been characterized to regulate both histones and non-histone proteins^4^,^6^,^9^. Currently, hundreds of lysine malonylated proteins have been discovered, and experimental evidence demonstrated that malonylation frequently occur together with other types of lysine PTMs such as succinylation and glutarylation, modifies both cytosolic and mitochondrial proteins, regulates the protein enzymatic activity, play a potential role in the regulation of metabolic pathways, and has been associated with type 2 diabetes^3^,^4^,^6^,^8^,^9^,^27^. In this regard, the identification of site-specific malonylation events in specific proteins is fundamental for further understanding the molecular mechanisms and regulatory roles of lysine malonylation.

In contrast with labor-intensive and time-consuming experimental efforts, computational prediction of protein malonylation sites can efficiently and rapidly provide useful information for further experimental manipulation. In this study, a new predictor Mal-Lys was developed for identifying the lysine malonylation sites in proteins. The benchmark dataset for training and testing was taken from a previously published large-scale experiment. Residue sequence order information, position-specific amino acid propensity and physicochemical properties have been used in feature construction. The mRMR method was used to select the optimal features. An online web-server was developed for the predictor which would facilitate the use for the biologists. The improved prediction of malonylation sites in proteins will be done when new malonylation sites data become available. We anticipate that Mal-Lys can be helpful for a better understanding of lysine malonylation.

## Methods

### Benchmark Dataset

The experimentally validated malonyllysine benchmark dataset used in this study was derived from a recently reported large-scale study^4^. There are 573 malonylated peptides (mainly lysine and cysteine) and 494 unique malonyllysine sites from 246 proteins were collected and the corresponding complete sequences were derived from the UniProt database^28^ (release 2015_01, http://www.uniprot.org/). To facilitate description later, for every peptide fragment with lysine (K) located at its center, it can be expressed as.


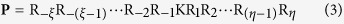


where the subscript ξ, η are integers, R_−ξ_ represents the ξ–th uptream amino acid residue from the center, R_*η*_ the η–th downstream amino acid residue, and so forth.

The average lengths of upstream and downstream are 4.804 ± 1.414 and 8.511 ± 0.707, respectively from the experimental peptides. So ξ = 6, η = 9 were adopted and the (ξ + 1 + η = 16)-tuple peptide can be further classified as positive peptide if K was malonylated, otherwise negative peptide if K was non-malonylated.

The benchmark dataset can be formulated as 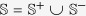 where 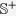 only contained the positive samples; 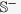
only contained the negative samples. If the upstream or downstream in a peptide was less than ξ or η, the lacking residues were filled with a dummy residue “*X*”. To reduce the redundancy and avoid homology bias which would overestimate the predictor, we removed those peptides that had ≥40% pairwise sequence identity to any other from the benchmark datasets.

Finally, we obtained the benchmark dataset which contained 458 (positive) + 3,974 (negative) peptide samples (see Table 1 and the Supplementary Information).

### Four metrics for measuring prediction quality

To illuminate the performance of the proposed predictor, four frequent measurements: sensitivity (*Sn*), specificity (*Sp*), accuracy (*Ac*), and Mathew correlation coefficient (*MCC*) were utilized.


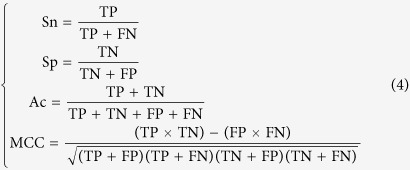


where TP (true positive) denotes the number of malonylated peptides correctly predicted, TN (true negative) the numbers non-malonylated peptides correctly predicted, FP (false positive) the non-malonylated incorrectly predicted as the malonylated peptides, and FN (false negative) the malonylated peptides incorrectly predicted as the non-malonylated peptides. Apart from the above criteria, the AUC value was used as an efficient indicator of robustness.

## Additional Information

**How to cite this article**: Xu, Y. *et al*. Mal-Lys: prediction of lysine malonylation sites in proteins integrated sequence-based features with mRMR feature selection. *Sci. Rep.* **6**, 38318; doi: 10.1038/srep38318 (2016).

**Publisher’s note:** Springer Nature remains neutral with regard to jurisdictional claims in published maps and institutional affiliations.

## Supplementary Material

Supplementary Dataset

## Figures and Tables

**Table 1:** The number of the benchmark dataset.

*No.*	*Positive*	*Negative*
Dataset S	458	3974
